
JOURNAL INFORMATION
==============================
NLM Title Abbreviation: ScientificWorldJournal
Journal ID: ScientificWorldJournal
NLM Journal ID: 101131163
Journal ID: TSWJ

The Scientific World Journal

ISSN: 2356-6140
EISSN: 1537-744X
Publisher: Wiley

ARTICLE INFORMATION
==============================
PMCID: PMC5936526
PMID: 15798883
DOI: 10.1100/tsw.2005.31
Article ID: 530216
Article version: 1
Subjects: Directions in Science

Profiling Heparan Sulfate Proteoglycans in Ovarian Carcinoma

Clamp Andrew R. *
Jayson Gordon C.
Cancer Research UK Department of Medical Oncology, Christie Hospital NHS Trust, Wilmslow Road, Withington, Manchester, M20 4BX, UK
*Andrew R. Clamp: aclamp@picr.man.ac.uk
Publication date: 2005
Electronic publication date: 2005 Mar 20
Volume: 5
First page: 230
Last page: 233
Received 2005 Feb 28; Revised 2005 Mar 4; Accepted 2005 Mar 4
Copyright: Copyright © 2005 Andrew R. Clamp and Gordon C. Jayson.
Copyright year: 2005
License: This is an open access article distributed under the Creative Commons Attribution License, which permits unrestricted use, distribution, and reproduction in any medium, provided the original work is properly cited.
License URL: https://creativecommons.org/licenses/by/3.0/

Keywords: heparan sulfate, proteoglycans, syndecan, glypican, perlecan, ovarian carcinoma, angiogenesis, prognostic factors

==============================
Directions in Science
*TheScientificWorldJOURNAL* (2005) 5, 230–233
ISSN 1537-744X; DOI 10.1100/tsw.2005.31

### Profiling Heparan Sulfate Proteoglycans in Ovarian Carcinoma

Andrew R. Clamp* and Gordon C. Jayson
*Cancer Research UK Department of Medical Oncology, Christie Hospital NHS Trust, Wilmslow Road, Withington, Manchester, M20 4BX, U.K.*

**E-mail**: aclamp@picr.man.ac.uk; Gordon.Jayson@christie-tr.nwest.nhs.uk

*Received February 28, 2005; Revised March 4, 2005; Accepted March 4, 2005; Published March 20, 2005*

**KEYWORDS**: heparan sulfate, proteoglycans, syndecan, glypican, perlecan, ovarian carcinoma, angiogenesis, prognostic factors

Heparan sulfate proteoglycans (HSPGs) are ubiquitously expressed cell surface and extracellular matrix (ECM) molecules consisting of a core protein covalently attached to the structurally complex glycosaminoglycan, heparan sulfate (HS).

While different HSPG family members synthesized by the same cell are likely to contain similar HS sequences[1], the HSPG core protein is critical in determining the localization within membrane microdomains or the ECM, and consequently the physiological role for HS[2]. There are two major classes of cell surface HSPGs: the syndecans and glypicans. The four syndecans are type I transmembrane proteins that carry three to five HS chains at their amino terminus. The family of six glypicans, however, are globular proteins attached to the cell by a glycosylphosphatidylinositol lipid anchor and contain two to four glycanation sites near their C-terminus resulting in close proximity of the decorating HS chains to the plasma membrane[3].

HSPGs are important coreceptors required for signaling by heparin-binding growth factors such as FGF-2 and VEGF, which recognize specific patterns of sulfation within the HS chains. The binding of cytokines to HSPGs regulates the response of cells/tissues to these growth factors and HSPGs, therefore, play key roles in many aspects of tumor biology including invasion, metastasis, and angiogenesis[4,5].

Several studies have examined the expression of single HSPG family members in cancer (summarized in 4,5). Until recently, the only HSPG to have been studied in detail in ovarian cancer was glypican-3, which may act as a negative regulator of growth in the ovary *in vivo* as GPC3 gene is transcriptionally silenced by hypermethylation in 30% of ovarian cancer cell lines[6] while the chromosomal region encompassing the GPC-3 locus is deleted in 30% of patients with sporadic ovarian cancer[7]. However, in a recent study published in *Clinical Cancer Research*[8], our group has compiled comparative immunohistochemical profiles of six HSPGs in normal ovary and ovarian cancer.

Using a panel of 12 normal ovaries, 10 benign, and 10 borderline tumors, as well as 115 ovarian carcinomas, we assessed the expression of syndecans-1–4, glypican-1, and perlecan, the major ECM HSPG. Syndecan-1 was absent from normal ovary, but present in the epithelial and stromal cells of a significant proportion of all ovarian tumor types where it was frequently concentrated at the most infiltrating area of the advancing invasive tumor front. Stromal syndecan-1 expression in carcinoma

*Corresponding author.
©2005 with author.

specimens was associated with shorter progression-free and overall survival on univariate analysis, although was not a significant prognostic factor after adjustment for disease stage, residual disease postsurgery, and patient performance status.

Normal or malignant epithelial tissue did not express glypican-1 or syndecans-2 and -3. However, stromal expression of these HSPGs was present in many specimens with staining intensity highest in carcinoma. Of note, expression of syndecan-3, conventionally thought to be a neuronal HSPG, was detected on large and small blood vessels in all tissue sections suggesting an important role in endothelial function. Perlecan staining was present in the epithelial basement membranes of all benign specimens, but was absent in 67% of carcinomas consistent with the adoption of an invasive phenotype.

These findings are important as they demonstrate marked changes in the HSPG expression profile of both epithelial and stromal tissue that occur early during ovarian tumorigenesis. Such changes may have a significant impact on the way in which these tissue compartments respond to heparin-binding growth factors.

Syndecan-1 has previously been demonstrated to have pleiotropic effects in tumorigenesis. *In vitro* expression of syndecan-1 is associated with maintenance of epithelial morphology and suppression of invasiveness[9], but its expression is essential for Wnt-1–induced mammary tumorigenesis in mice[10]. Previous immunohistochemical studies have also generated contradictory results[11,12,13,14]. Our study, however, indicates that stromal syndecan-1 expression is associated with tumor progression and emphasizes the importance of the stromal compartment in ovarian tumorigenesis. Notably, stromal syndecan-1 expression is also induced in human breast carcinoma[13,15] and has been demonstrated to promote carcinoma cell proliferation in *in vitro* coculture models[15] reinforcing the signficant role of syndecan-1 in the functional interplay between stroma and carcinoma.

However, to completely understand the roles that HSPGs play in tumorigenesis and open up new therapeutic avenues, we must appreciate not just changes in core protein expression, but also the distribution of the HS epitopes recognized by heparin-binding growth factors. Preliminary work has shown that the composition of cell surface HS changes with tumor progression[16] and the expression of the newly identified HS-editing enzymes, H-sulf-1 and -2, are downregulated in several tumor types[17,18].

Recently, two new techniques have been developed that may address this. First, the application of phage display technology has generated a panel of antibodies that are capable of discriminating distinct HS epitopes[19,20] in tissue sections, although the precise sequences recognized by these antibodies need to be further defined. Second, a unique molecular probe (FGF Receptor 1 conjugated to alkaline phosphatase: FR1-AP) has been constructed that can be employed as a potential method for mapping bioactive HS sequences capable of supporting FGF-2 signaling *in vivo*[21]. FR1-AP only recognizes a subset of tissue-bound FGF-2 suggesting that only at these sites are the FGF-2/HS complex able to support formation of an active trimolecular signaling unit. We have recently shown that within ovarian serous adenocarcinoma, FRAP binds almost exclusively to endothelial cell membranes probably via HS expressed on syndecan-3 providing *in situ* evidence for the importance of HS in ovarian cancer angiogenesis[22].

The construction of an HSPG profile of ovarian cancer has demonstrated marked alterations in the expression of these important molecules in both epithelial and stromal compartments that occur early in tumorigenesis[8]. It is an important step towards developing a full “glycoprofile”[5] of cancer that will direct the emerging field of oligosaccharide therapeutics towards more effective cancer therapies.

### REFERENCES

1. Zako, M., Dong, J., Goldberger, O., Bernfield, M., Gallagher, J.T., and Deakin, J.A. (2003) Syndecan-1 and -4 synthesized simultaneously by mouse mammary gland epithelial cells bear heparan sulfate chains that are apparently structurally indistinguishable. *J. Biol. Chem.* **287**, 13561–13569.
2. Gallagher, J.T. (2001) Heparan sulfate: growth control with a restricted sequence menu. *J. Clin. Invest.* **108**, 357–361.
3. Bernfield, M., Gotte, M., Park, P.W., Reizes, O., Fitzgerald, M.L., Lincecum, J., and Zako, M. (1999) Functions of cell surface heparan sulfate proteoglycans. *Annu. Rev. Biochem.* **68**, 729–777.
4. Blackhall, F.H., Merry, C.L.R., Davies, E.J., and Jayson, G.C. (2001) Heparan sulphate proteoglycans and cancer. *Br. J. Cancer* **85**, 1094–1098.
5. Sasisekharan, R., Shriver, Z., Venkataraman, G., and Narayanasami, U. (2002) Roles of heparan sulphate glycosaminoglycans in cancer. *Nat. Rev. Cancer* **2**, 521–528.
6. Lin, H., Huber, R., Schlessinger, D., and Morin, P.J. (1999) Frequent silencing of the GPC 3 gene in ovarian cancer cell lines. *Cancer Res.* **59**, 807–810.
7. Choi, C., Cho, S., Horikawa, I., Berchuck, A., Wang, N., Cedrone, E., Jhung, S.W., Lee, J.B., Kerr, J., Chenevix-Trench, G., Kim, S., Barrett, J.C., and Koi, M. (1997) Loss of heterozygosity at chromosome segment Xq25-26.1 in advanced human ovarian carcinomas. *Genes Chromosomes Cancer* **20**, 234–242.
8. Davies, E.J., Blackhall, F.H., Shanks, J.H., David, G., McGown, A.T., Swindell, R., Slade, R.J., Martin-Hirsch, P., Gallagher, J.T., and Jayson, G.C. (2004) Distribution and clinical significance of heparan sulfate proteoglycans in ovarian cancer. *Clin. Cancer Res.* **10**, 5178–5186.
9. Inki, P. and Jalkanen, M. (1996) The role of syndecan-1 in malignancies. *Ann. Med.* **28**, 63–67.
10. Liu, B.Y., Kim, Y.C., Leatherberry, V., Cowin, P., and Alexander, C.M. (2003) Mammary gland development requires syndecan-1 to create a β-catenin/TCF-responsive mammary epithelial subpopulation. *Oncogene* **22**, 9243–9253.
11. Anttonen, A., Kajanti, M., Heikkila, P., Jalkanen, M., and Joensuu, H. (1999) Syndecan-1 expression has prognostic significance in head and neck carcinoma. *Br. J. Cancer* **79**, 558–564.
12. Kumar-Singh, S., Jacobs, W., Dhaene, K., Weyn, B., Bogers, J., Weyler, J., and Van Marck, E. (1998) Syndecan-1 expression in malignant mesothelioma: correlation with cell differentiation, WT1 expression and clinical outcome. *J. Pathol.* **186**, 300–305.
13. Stanley, M.J., Stanley, M.W., Sanderson R.D., and Zera, R. (1999) Syndecan-1 expression is induced in the stroma of infiltrating breast carcinoma. *Am. J. Clin. Pathol.* **112**, 377–383.
14. Wiksten, J.P., Lundin, L., Nordling, S., Lundin, M., Kokkola, A., von Boguslawski, K., and Haglund, C. (2001) Epithelial and stromal syndecan-1 expression as predictor of outcome in patients with gastric cancer. *Int. J. Cancer* **95**, 1–6.
15. Maeda, T., Alexander, C.M., and Friedl, A. (2004) Induction of syndecan-1 expression in stromal fibroblasts promotes proliferation of human breast cancer cells. *Cancer Res.* **64**, 612–621.
16. Jayson, G.C., Lyon, M., Paraskeva, C., Turnbull, J.E., Deakin, J.A., and Gallagher, J.T. (1998) Heparan sulfate undergoes specific structural changes during the progression from human colon adenoma to carcinoma in vitro. *J. Biol. Chem.* **273**, 51–57.
17. Lai, J., Chien, J., Staub, J., Avula, R., Greene, E.L., Matthews, T.A., Smith, D.I., Kaufmann, S.H., Roberts, L.R., and Shridhar, V. (2003) Loss of HSulf-1 up-regulates heparin-binding growth factor signaling in cancer. *J. Biol. Chem.* **278**, 23107–23117.
18. Lai, J.P., Chien, J., Strome, S.E., Staub, J., Montoya, D.P., Greene, E.L., Smith, D.I., Roberts, L.R., and Shridhar, V. (2004) HSulf-1 modulates HGF-mediated tumor cell invasion and signaling in head and neck squamous carcinoma. *Oncogene* **23**, 1439–1447.
19. ten Dam, G.B., van de Westerlo, E.M., Smetsers, T.F., Willemse, M., van Muijen, G.N., Merry, C.L., Gallagher, J.T., Kim, Y.S., and van Kuppevelt, T.H. (2004) Detection of 2-O-sulfated iduronate and N-acetylglucosamine units in heparan sulfate by an antibody selected against acharan sulfate (IdoA2S-GlcNAc)n. *J. Biol. Chem.* **279**, 38346–38352.
20. Dennissen, M.A., Jenniskens, G.J., Pieffers, M., Versteeg, E.M., Petitou, M., Veerkamp, J.H., and van Kuppevelt, T.H. (2002) Large tissue-regulated domain diversity of heparan sulfates demonstrated by phage display antibodies. *J. Biol. Chem.* **277**, 10982–10986.
21. Chang, Z., Meyer, K., Rapraeger, A., and Friedl, A. (2000) Differential ability of heparan sulfate proteoglycans to assemble the fibroblast growth factor receptor complex *in situ*. *FASEB J.* **14**, 137–144.
22. Whitworth, M.K., Backen, A.C., Clamp A.R., Wilson, G., McVey, R., Friedl, A., Raprager, A.C., David, G., McGown, A., Slade, R.J., Gallagher, J.T., and Jayson, G.C. (2005) Regulation of FGF2 activity by human ovarian cancer tumor endothelium. *Clin. Cancer Res.*, accepted for publication.

---

**This article should be referenced as follows:**

Clamp, A.R. and Jayson, G.C. (2005) Profiling heparan sulfate proteoglycans in ovarian carcinoma. *TheScientificWorldJOURNAL* **5**, 230–233.

**Handling Editor:**

Martin Gotte, Principal Editor for *Cell Biology* and Editorial Board Member for *Biochemistry* — domains of *TheScientificWorldJOURNAL*.

---

### BIOSKETCHES

**Dr Andrew R. Clamp,** MSc MRCP, holds a Cancer Research UK McElwain Research Fellowship for Clinician. He is undertaking a PhD exploring the interactions of endostatin and heparan sulfate proteoglycans. His current clinical interests include Phase I trials and ovarian cancer.

**Dr. Gordon C. Jayson,** FRCP PhD, is a Senior Lecturer and Consultant in Medical Oncology. His research interests include ovarian cancer, phase I trials with a focus on angiogenesis and the investigation of the heparan sulfate growth factor axis in the laboratory. He is the recipient of the BUPA Foundation Best Emerging Researcher Award 2004.
